# Senescence accelerated mouse-prone 8: a model of neuroinflammation and aging with features of sporadic Alzheimer’s disease

**DOI:** 10.1093/stmcls/sxae091

**Published:** 2025-01-15

**Authors:** Jun Ong, Kazunori Sasaki, Farhana Ferdousi, Megalakshmi Suresh, Hiroko Isoda, Francis G Szele

**Affiliations:** Department of Physiology, Anatomy and Genetics, University of Oxford, Oxford OX13QX, United Kingdom; Alliance for Research on the Mediterranean and North Africa (ARENA), University of Tsukuba, Tsukuba, Ibaraki 305-8572, Japan; Alliance for Research on the Mediterranean and North Africa (ARENA), University of Tsukuba, Tsukuba, Ibaraki 305-8572, Japan; Institute of Life and Environmental Sciences, University of Tsukuba, Japan1-1-1 Tennodai, Tsukuba, Ibaraki 305-8572, Japan; Department of Physiology, Anatomy and Genetics, University of Oxford, Oxford OX13QX, United Kingdom; Alliance for Research on the Mediterranean and North Africa (ARENA), University of Tsukuba, Tsukuba, Ibaraki 305-8572, Japan; Alliance for Research on the Mediterranean and North Africa (ARENA), University of Tsukuba, Tsukuba, Ibaraki 305-8572, Japan; Institute of Life and Environmental Sciences, University of Tsukuba, Japan1-1-1 Tennodai, Tsukuba, Ibaraki 305-8572, Japan; Department of Physiology, Anatomy and Genetics, University of Oxford, Oxford OX13QX, United Kingdom

**Keywords:** Alzheimer’s, SAMP8, aging, neuroinflammation, mouse models

## Abstract

The large majority of Alzheimer’s disease (AD) cases are sporadic with unknown genetic causes. In contrast, only a small percentage of AD cases are familial, with known genetic causes. Paradoxically, there are only few validated mouse models of sporadic AD but many of familial AD. Senescence accelerated mouse-prone 8 (SAMP8) mice are a model of accelerated aging with features of sporadic AD. They exhibit a more complete suite of human AD-relevant pathologies than most familial models. SAMP8 brains are characterized by inflammation, glial activation, b-amyloid deposits, and hyperphosphorylated Tau. The excess amyloid deposits congregate around blood vessels leading to vascular impairment and leaky BBBs in these mice. SAMP8 mice also exhibit neuronal cell death, a feature not typically seen in models of familial AD. Additionally, adult hippocampal neurogenesis is decreased in SAMP8 mice and correspondingly, they have reduced cognitive ability. In line with this, hippocampal LTP is significantly compromised in SAMP8 mice. No model is perfect and SAMP8 mice are limited by the lack of clarity about their genomic differences from control Senescence Accelerated Mouse-Resistant 1 (SAMR1) mice although their transcriptomics changes are being revealed. To further complicate matters, multiple substrains of SAMP8 mice have emerged over the years, sometimes making comparisons of studies difficult. Despite these challenges, we argue that SAMP8 mice can be useful for studying AD-relevant symptoms and propose important experiments to strengthen this already useful model.

## Graphical Abstract

**Graphical Abstract:**
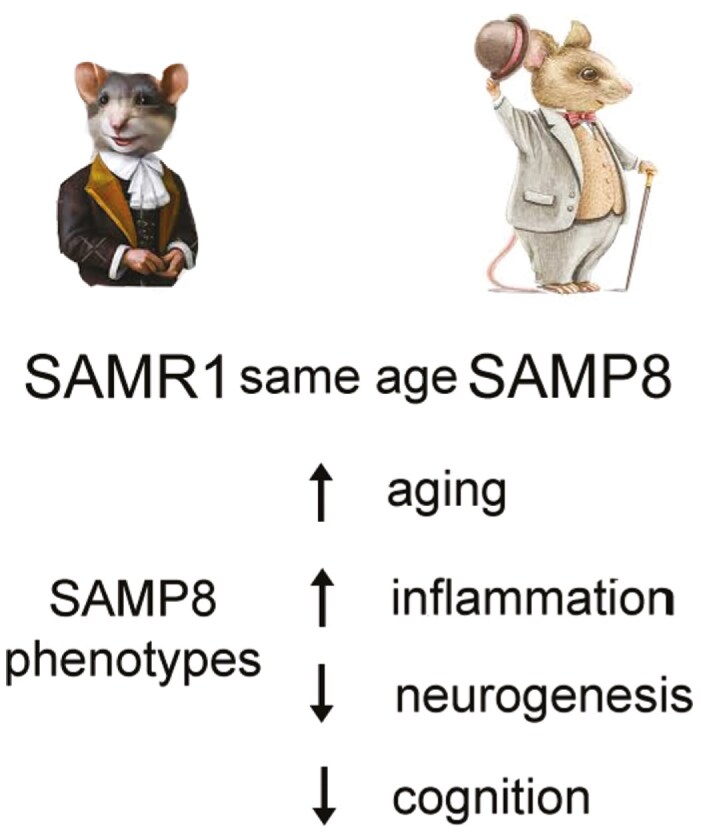

## Introduction

The Senescence Accelerated Mouse (SAM) models resulted from inbreeding AKR/J mice and selecting mice that aged prematurely. This gave rise to the SAM-prone (SAMP) mouse series while SAM-resistant (SAMR) series were derived from mice showing normal aging.^1^ Of particular interest, SAMP8 mice showed cognitive defects as early as 16 weeks of age. Two separate studies also indicate that they exhibit hyperphosphorylation of Tau.^2,3^ In the last decade, many studies have revealed the strengths of SAMP8 mice as a model for sporadic Alzheimer’s disease (AD) and have used it to test therapeutic interventions for AD-related behavioral defects and histopathology. Previous reviews of the SAMP8 model have characterized the behavioral changes such as spatial learning defects in multiple water maze tasks including the Morris water maze (MWM). This has been extensively reviewed by Cheng et al, and the performance of SAMP8 mice of different ages compared across studies.^4^ SAMP8 mice have deficits in object recognition, active and avoidance tasks, and fear-conditioning, and these deficits worsen with age.^4^ We also have shown that SAMP8 compared to SAMR1 mice have significant reductions in memory as measured by the MWM.^5,6^ Several of these behavioral tasks, such as the MWM and fear conditioning, involve the hippocampus and are regulated in part by adult hippocampal neurogenesis (AHN), which is reduced in older SAMP8 mice.^7^

In this review, we focus on studies that have characterized the neuropathological traits of the SAMP8 model, including changes to neuronal morphology, survival, inflammation, and neurogenesis. We also provide a summary of the genetic and transcriptomic changes in the SAMP8 brain. We show that various studies with differing methodologies make it difficult to thoroughly understand the changes that occur during the lifespan of the SAMP8 mouse, both pre- and post-symptomatically. For example, a study has shown that as early as 6 months SAMP8 mice exhibit Ab granules,^8^ but how similar this is to Ab plaques is unclear. Finally, we highlight key areas in which the literature is lacking and propose a detailed study of these characteristics to better understand SAMP8 mice at the molecular level. We hope that this will generate new insights into AD and its pathogenesis, identify novel targets which are druggable or can be treated with nutraceuticals, and lastly provide the field with molecular endpoints to show how well new therapies are working.

### Inflammation in SAMP8 mice

SAMP8 mice show a progressive decline in immune responses, which likely contributes to their neuroinflammatory profile and cognitive impairment. As early as 5 weeks old, these mice exhibit significantly reduced natural killer cell activity and impaired antibody responses compared to age-matched SAMR1 mice.^9^ This decline in immune activity is attributed to splenic CD4+ T-cell dysfunction at 2 months^9^ and altered splenic B-cell subpopulations at 6 months.^10^ Additionally, the biosynthesis of specialized pro-resolving mediators (SPMs), the key regulators in resolving inflammation, is insufficient in 9-month-old SAMP8 mice brains compared to normal aging SAMR1 mice.^11^ Interestingly, the SPM biosynthetic enzyme leukocyte-type 12-lipoxygenase (L12-LOX) co-localizes with Aβ clusters and correlates positively with phosphorylated tau levels at Ser202/Thr205 in the brains of aged SAMP8 mice, but not in SAMR1.^11^ This progressively impaired neuroimmune response might partly explain the variability in pro-inflammatory cytokine levels observed in normal and inflammation-induced SAMP8 mice. SAMP8 mice also model oxidative stress, with 6- and 10-month-old mice having increased levels of reactive oxygen species (ROS), and decreased superoxide dismutase (SOD) activity.^12,13^

IL-1β and TNF-α mRNA levels are significantly higher in the hippocampus of 4- and 8-month-old SAMP8 compared to SAMR1 mice.^14^ Similarly, microarray analysis of cortical samples from 16-week and 1-year-old mice showed elevated pro-inflammatory cytokine gene expression in SAMP8 compared to SAMR1 mice.^15^ A study in 10-month-old mice found that IL-1β expression was markedly higher in SAMP8 mice across several brain regions, including the hippocampus, hypothalamus, and brain stem, with the largest increase in the hippocampus.^16^ Significant differences in TNF-α and IL-6 have been found in SAMP8 brains.^16,17^ Interestingly, the authors observed no significant changes in cytokine expression in response to endotoxin LPS stimulation between SAMP8 and SAMR1 at 5 months. Another study highlighted the vulnerability of the hippocampus to systemic LPS stimulation, demonstrating that cytokine activation induced by LPS at 6 months was absent at 12 months, reflecting an age-dependent impairment in the inflammatory response.^18^

Microglial activation plays a crucial role in this exaggerated inflammatory response in SAMP8s. Microglia in SAMP8 mice display an activated globular morphology in both control and LPS-challenged conditions and produce higher nitric oxide levels compared to SAMR1, independent of LPS exposure.^18^ Enhanced microglial activation is evident from stronger F4/80 staining in SAMP8s, with an age-dependent increase. Additionally, MHC class II, a marker of microglial activation, was significantly elevated in the hippocampus of both 2- and 9-month-old SAMP8s compared to age-matched SAMR1s and showed an age-dependent increase within SAMP8 mice.^11^ However, Fernandez et al reported no significant age-dependent increase in Iba1-stained microglial cells, suggesting the need for further investigation.^19^

This accumulating evidence in SAMP8 mice indicates that aging amplifies the effects of neuroinflammation on cognitive function, and inflammation exacerbates aging-related cognitive decline. Understanding these interconnections may provide insights into therapeutic targets to mitigate age-related neuroinflammation and cognitive impairment.

### Alzheimer’s like features of histopathology in SAMP8 mice

SAMP8 mice are a powerful model for sporadic AD as they exhibit several features of inflammation and histopathology that are characteristic of AD. These include microglial activation, amyloidosis, and Tau hyperphosphorylation.^4,17^ SAMP8 mice also exhibit greater neuronal cell death than control SAMR1s, a feature not typically seen in familial AD (FAD) models.^4^ Hippocampal neuronal loss is detected as early as 3 months in SAMP8s.^3^ By 8 months, neuronal numbers are reduced in the dentate gyrus (by 20%), CA1 (by 20%) and CA3 (by 16%).^20^ Neuronal loss in SAMP8 mice is also seen in the cerebral cortex and other brain regions.^21-23^

SAMP8 mice exhibit other classical features of AD such as Tau hyperphosphorylation, with several studies showing increased levels of various phosphorylated Tau (pTau) species in SAMP8, including pThr231,^3,24^ pSer214,^3^ pSer396, and pSer199.^24^ Moreover, SAMP8 mice also show increased pTau immunoreactivity in neuronal cell bodies.^3,21,24,25^ Despite this, characterization of these Tau aggregates is still lacking. As such, the field will benefit from ultrastructural characterization of these Tau aggregates to determine the nature of tauopathy in the SAMP8 model.

These changes in Tau are accompanied by microglial inflammation, reactive astrocytes with high levels of GFAP, and disrupted neuronal homeostasis.^4,26^ Five- and 10-month-old SAMP8 mice have a larger number of activated microglia in the cerebral cortex than SAMR1s signifying inflammation.^21,27^ Sureda et al also found reactive astrocytes surrounding blood vessels in 5-month-old SAMP8 mice. Thus, the timing of neuropathology progresses at a well-understood pace in SAMP8s and allows dissection of the timing of neuropathology at specific ages.

SAMP8 mice overproduce Aβ_1-42_ compared to SAMR1.^17,28^ Studies characterizing amyloid pathology in SAMP8s using anti-Aβ antibodies show the presence of Aβ “granules” in the hippocampus of SAMP8 mice at 3 months^8^ and 10 months,^29^ with increasing amounts seen with age.^8^ Further characterization of these granules shows colocalization with Tau.^30^ Furthermore, Congo Red-positive plaques have been seen in SAMP8s at 9 months of age, albeit at a lower amyloid load than APP/PS1 transgenic mice.^31^ Similarly stained plaques were also seen in SAMP8s but not SAMR1s at 8 months of age.^32^ However, others have reported not finding amyloid plaques in SAMP8s,^3,22^ or only found Aβ deposits.^8,33,34^

SAMP8 mice have excess Ab and amyloid deposits, especially around blood vessels leading to vascular impairment and leaky BBBs.^4,17,26^ The protease inhibitor SERPINA3 colocalizes with accumulated amyloid peptides in AD brains.^35^ Interestingly, SAMP8 mice express Neuroserpin A3 in their brainstem, and polymorphic SERPINA3 I308T (rs142398813) prolonged toxic oligomeric Aβ42 formation and neuronal cell death in comparison to wild-type SERPINA3 protein.^36^ For these reasons, while SAMP8 mice have been considered a model of sporadic Alzheimer’s disease^34,37^ and recapitulate some of the amyloid and Tau pathologies of AD, future work in SAMP8 mice needs to characterize the nature of tauopathy and amyloid deposits as these hallmarks of AD are still contested.

### Hippocampal neurogenesis in SAMP8 mice

Alzheimer’s disease frequently commences in the hippocampus, a major site that regulates learning and memory. Fortuitously, the subgranular zone of the dentate gyrus is a major site of adult hippocampal neurogenesis (AHN) which can be detected throughout life.^38-41^ Stimulating AHN in rodents improves memory and pattern separation, whereas reduced hippocampal neurogenesis decreases memory functions.^42-46^ One of the ways the rate of AHN is regulated is via the elimination of newborn neurons; many newborn neurons undergo cell death within 4 days, the interval between 1W and 4W is also crucial and thereafter, most cells that survive will continue to survive past 11 month in mice.^47-49^

Most studies show decreased neurogenesis in SAMP8 mice compared to SAMR1 controls^7,20,50-53^ (Table 1), while some identify increased neurogenesis in younger SAMP8s as compared to SAMR1s.^7^ This is similar to FAD mouse models, and it is proposed that the initial increase in neurogenesis is eventually insufficient to replace the loss. Across the studies, SAMP8s of different ages are used, from as early as 1M to 10M of age. Additionally, while most studies use the neuroblast marker DCX as a marker for neurogenesis, some studies include BrdU regimens to measure changes in dividing neural stem cells (BrdU^+^GFAP^+^ or BrdU^+^Sox2^+^), dividing neuroblasts (BrdU^+^DCX^+^). Others also include BrdU together with treatment regiments and use BrdU^+^NeuN^+^ to measure the number of neurons that matured that had been generated during the time of treatment. Together, these studies identify the defects in neurogenic subpopulations of the hippocampus and the ages at which these populations are affected. This, in turn, informs research on compounds that can ameliorate these defects.

**Table 1.** SAMP8 mice have deficits in neurogenesis, as seen from the reduced BrdU + DCX+ and DCX+ population reported by multiple studies (changes to neurogenesis in SAMP8). However, some studies have reported increases in DCX+ cells in younger SAMP8 mice (<6 months). As such, decreases in neurogenesis appear to be limited to the neuroblasts and mature neurons, with studies looking at the proliferation of neural stem cells showing no significant changes. In general, SAMP8 mice show increased density of microglia and astrocytes, in both cell numbers and immunoreactive areas (changes to glia in SAMP8). One other study has also shown decreases in the ramification of microglia.^27^ In almost all studies, SAMP8s show cognitive decline with the behavioral tests used, including the Y-maze, Morris Water Maze (MWM), Novel Object Recognition Test (NORT), and passive avoidance.

Changes to neurogenesis in SAMP8			
Neurogenesis measurement	Age of mice	SAMP8 vs R1	Publications
BrdU^+^DCX^+^	<6 months	Decreased	^51^
	6 to 8 months	Decreased	^54^
DCX^+^	<6 months	Decreased	^3^
		Increased	^7,50^
	>8 months	Decreased	^52,53^
BrdU^+^NeuN^+^	<6 months	Decreased	^7,50^
	>8 months	Decreased	^7^
Changes to glia in SAMP8			
Neuroinflammation measurement	Age of mice	SAMP8 vs R1	Publications
Iba1 area (Cortex)	6-8 months	Decreased	^55^
	>8 months	Decreased	^55^
		Increased	^56,57^
Iba1 area (Hippocampus)	6-8 months	Increased	^58^
	>8 months	Increased	^57,59,60^
Gfap area (Cortex)	6-8 months	Increased	^3,61,62^
	>8 months	Increased	^3,57^
Gfap area (Hippocampus)	<6 months	Increased	^63^
	6-8 months	Increased	^55,61,62,64,65^
	>8 months	Decreased	^55,63^
		Increased	^57,59,66^
Iba1 density/number (Cortex)	>8 months	Increased	^56^
Iba1 density/number (Hippocampus)	6-8 months	Increased	^53,67-73^
	>8 months	Decreased	^72^
		Increased	^53,73^
Gfap density/number (Hippocampus)	6-8 months	Increased	^33,68,69,71^
	>8 months	Decreased	^52^
		Increased	^73^
Gfap intensity (Cortex)	>8 months	Increased	^74^
Gfap intensity (Hippocampus)	6-8 months	Increased	^75,76^
	>8 months	Increased	^74^
Changes to behavior in SAMP8			
Behavioral test	Age of mice	SAMP8 v.s. R1	Publications
Y-maze	6M	Time in new arm↓, Time in known arm↑	^3^
Y-maze	9M		^3^
MWM	6M	Increased escape latency	^50^
MWM	4M	Increased escape latency	^77^
NORT	10M	Decreased short- and long-term memory	^52^
MWM	4M	Increased escape latency	^6^
Passive avoidance	8M	Shorter memory retention	^53^

**Figure 1.**
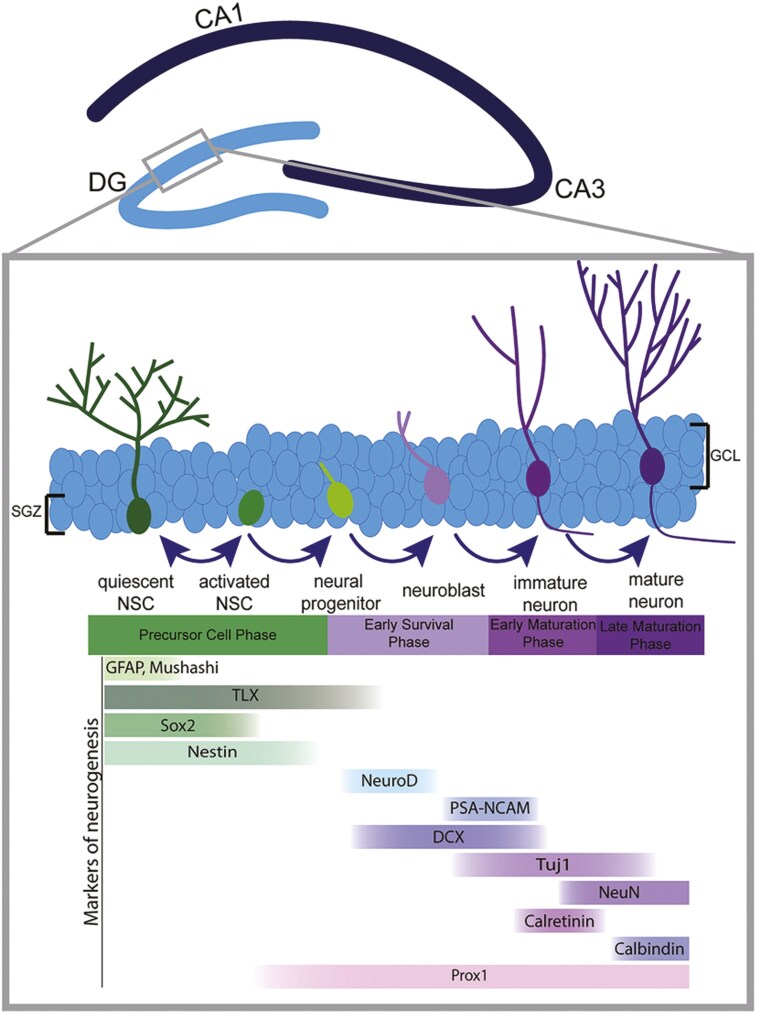
AHN in the subgranular zone showing lineage progression from quiescent neural stem cells all the way through to mature neurons. Note that each stage is labeled by multiple markers and the markers gradually change their expression levels. Reproduced with permission from Babcock et al (2021).^41^

**Figure 2.**
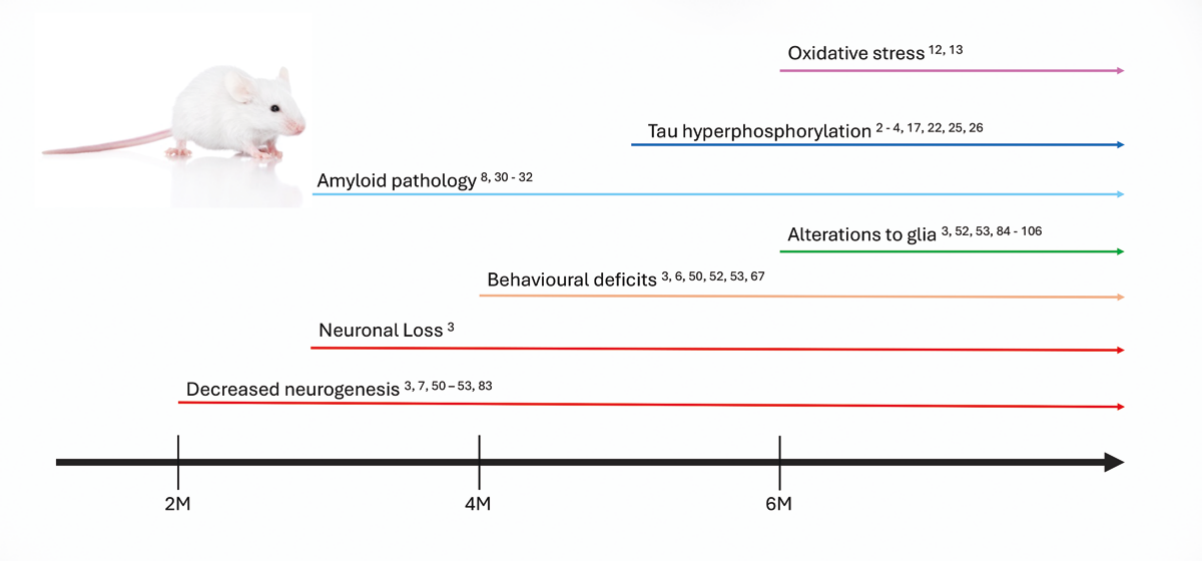
Disease progression in SAMP8 mice. Note that the first detected phenotype is decreased (hippocampal) neurogenesis. Also of importance is that increased expression of neuroinflammatory cytokines is detected before Tau hyperphosphorylation and amyloid deposition.

Much work in mice has revealed the progenitor subtypes and molecular regulation of AHN in mammals and several papers have established its function in SAMP8 mice (Figures 1 and 2 and Table 1). Using BrdU (a marker of the S-phase of the cell cycle) with SOX2, GFAP, DCX, and NeuN, Diaz-Moreno et al showed that although early progenitors are unaffected, the maturing DCX^+^ neuroblast population is significantly reduced in SAMP8 mice.^50^ In line with this, hippocampal LTP is significantly compromised in SAMP8 mice.^78^ Another study examined changes in neurogenesis of SAMP8 mice at 5 and 10 months.^7^ However the study of SAMP8 at 5 months is after they start showing cognitive defects (16W). It may be important to establish an earlier “baseline” before the SAMP8 exhibits histopathological defects. We have shown that pharmacological stimulation of adult hippocampal neurogenesis is associated with improved memory in SAMP8 mice, which has important translational relevance.^5^

Importantly, multiple studies have shown that AHN can persist in aged humans and that this is altered in AD.^40,79^ This highlights the relevance of SAMP8 as a model of sporadic AD and use for the discovery of pharmacological interventions that increase AHM.

### Galectin-3 in AD and mouse models

One of the most consistent hallmarks of AD is inflammation, yet how inflammation exacerbates or possibly gives rise to AD is an important open question. Galectins function as inflammation regulators and Gal-3 has emerged as an important potentially upstream regulator of AD.^80^ While all members of the galectin family have a c-terminal carbohydrate recognition domain (CRD), Galectin-3 (Gal-3) is the only chimeric member of the family; its n-terminal protein-binding domain allows it to oligomerize with other Gal-3 molecules.^81^ We previously reviewed Gal-3 and its role in neurogenesis and neurological conditions, including AD.^80^

Gal-3 is usually pro-inflammatory, including in brain injury, where it mediates microglial activation.^82^ Importantly, Gal-3 is elevated in the serum^83,84^ and cerebrospinal fluid^85^ of AD patients. In mouse AD models, Gal-3 is localized to Aβ-plaques, specifically in Aβ-plaque-associated microglia.^86,87^ The involvement of Gal-3 in AD is further bolstered by SNPs found in the *LGALS3* gene which increase the risk of developing AD.^86^ Gal-3 is a druggable target with small molecule inhibitors in clinical trials for inflammatory diseases and AD. A better understanding of the role of Gal-3 in AD can inform us when Gal-3 inhibitors should be used. We should aim to specify whether Gal-3 levels are increased before or after pathological symptoms (ie, Braak I-II, III-IV, or further) which may help confirm causation.

Two complementary studies examined the role of Gal-3 in FAD models; in 5xFAD and APP/PS1 mice. Gal-3 expression in the brain increased in an age-dependent manner.^86,87^ Functional studies showed that endogenous Aβ-oligomerisation was decreased in Gal-3 knockouts.^86,87^ Both studies showed that Gal-3 physically interacts with TREM2 a major risk factor for AD and Boza-Serrano et al showed that this interaction required the CRD of Gal-3.^86^ Gal-3 activation of microglia was TREM2-dependent.^87^ In 5xFAD/Gal-3KO brain slices, recombinant Gal-3 was shown to bind to TREM2^+^/Iba1^+^, but not TREM2^−^/Iba1^+^ microglia. Expression of disease-associated microglia (DAM)-genes was also reduced in 5xFAD/Gal3-KO.^86^ Plaque-associated microglia expressed not only Gal-3 but also Clec7a, a major DAM gene.^86^ Crucially, in WT mice, injections of recombinant human Gal-3 along with Aβ-fibrils into the hippocampus, increased the formation of Aβ plaques compared to Ab injections alone.^86^ These studies strongly suggested that Gal-3 could induce AD histopathology symptoms, but they do not address Gal-3 function in sporadic AD.

The expression of Gal-3 in SAMP8 mice is, to the best of our knowledge, unknown. We believe that comparing the expression of Gal-3 in the hippocampus and pre-frontal cortex of SAMP8 mice with that of age-matched SAMR1 controls will allow a better understanding of its role in AD. As with the other parameters, we hope to characterize the expression before the onset of symptoms, and also in late-stage diseased mice, where we expect to find Gal-3 expressed in a similar manner to human AD and FAD mice. To further the experiments in FAD models, we believe that investigating the ability of Gal-3 inhibitors to prevent Aβ-plaque formation will tell us whether there is potential for Galectin-3 as a target in AD.

### Molecular genetics of SAMP8 mice

Even though SAMP8 mice have been used for several decades, the literature is relatively sparse regarding their transcriptomes.^88^ Exome sequencing has been used in SAMP8s,^89,90^ but these may not be as informative regarding AD as they did not look at brain-specific gene expression. Delerue et al identified single nucleotide variants unique to SAMP8 in genes associated with a wide range of functions, including cytokine activity and axonogenesis.^89^ Tanisawa et al found Aifm3 as a mutation unique to SAMP8 among other SAMP strains.^90^ while its function is not fully known, overexpression of Aifm3 induces apoptosis in HEK293 and Aifm3 is also related to Aifm1 which is essential for nuclear disassembly in apoptotic cells, suggesting this mutation could contribute to mitochondrial defects in SAMP8. Bulk RNAseq of SAMP8 mice was performed in the context of stress (chronic noise exposure) but without SAMR1 controls.^88^ In this study, negative controls were non-exposed SAMP8s at 4M and positive controls were 8M SAMP8s. As mentioned, SAMP8s already show behavioral pathologies at 4M and perhaps even at 3M using more sensitive tests. While the authors found that chronic stress increased the expression of AD-associated genes, the lack of SAMR1 controls makes it impossible to compare at younger ages as well, which the researchers highlighted.^88^

We previously published work using microarray analysis of gene expression changes in SAMP8 vs SAMR1.^77,91^ Microarray analysis of cortical samples comparing both young (16 weeks) and aged (1 year) SAMP8 and SAMR1 mice revealed that neurodevelopmental processes were the most significantly affected biological functions, both in interstrain comparisons at early and late stages, as well as in intrastrain comparisons (1 year vs 16 weeks).^15^ Cell type enrichment analysis showed that neuron-specific genes were predominantly affected at both early and late stages of aging in SAMP8. In contrast, genes related to myelinating oligodendrocytes were significantly affected in young SAMP8 mice compared to age-matched SAMR1. Finally, dysregulation of synaptic function and lipid metabolism was observed early in SAMP8 mice and progressively worsened with age in both strains.^15^ In another study we used sugarcane top ethanolic extract (STEE) and showed 119 genes regulated in the same direction in the cortex of STEE-treated SAMP8 mice and SAMR1 mice (vs SAMP8 control), suggesting that STEE treatment could ameliorate some effects of aging.^77^ Furthermore, in a meta-analysis of microarray studies comparing the effects of 3,4,5-tricaffeolyquinic acid on neurogenesis, we integrated datasets from human in vitro and mouse in vivo studies, validating results across the studies.^91^ Our meta-analysis revealed that TCQA treatment resulted in DE genes enriched in terms related to cell cycle, VEGFA-VEGFR2, and BMP signaling.^91^ Trigonelline, a plant alkaloid commonly found in coffee beans, also modulated the accelerated aging-associated gene expression profile in the hippocampus of 16-week-old SAMP8 mice, particularly mitigating aging-induced neuroinflammation and synaptic dysregulation.^92^ These studies highlight the utility of SAMP8 mice for the discovery of pathways through which nutraceutical compounds act to improve cognitive function.

Recently, papers comparing single-cell RNA sequencing (scRNAseq) transcriptomics between AD patients and healthy aged controls have been published.^93^ These give in-depth insight into potential molecular mechanisms of AD at a level of granularity previously unseen. With the previous approaches of bulk RNAseq or qPCR, differentially expressed genes (DEGs) in minority cell populations may not be captured. An example that is especially pertinent to AD is microglia, which are important in specific types of neuroinflammation such as disease associated microglia (DAM).^94^ DEGs that are regulated in opposite directions in different cell types may also not be reflected in bulk RNAseq. In both these cases, scRNAseq will reveal differences and can give novel insights into how different cell populations are responding in AD. For example, a study using single nucleus RNA sequencing (snRNAseq) showed strong upregulation of the key AD gene APOE in microglia, but an opposite effect in astrocytes.^95^ Notably, a recent study by Ximerakis et al used scRNAseq of the whole mouse brain and compared gene expression in young (2-3M) and aged (21-23M) mice.^96^ This is exciting as it will allow the comparison of the SAMP8 to the aged mice. Additionally, their analysis shows that “aging, rather than inducing a universal program, drives a distinct transcriptional course in each cell population,” giving strength to our argument that bulk RNAseq may lose these details.

It will be fascinating to examine aging transcriptomics in different brain regions, especially the neurogenic niches. Recently a landmark study using single nuclear RNAseq demonstrated that cells in the human hippocampus at various ages have features of young newborn neurons, and some of these were proliferative, giving credence to the argument that AHN does indeed persist in humans.^97^ Recent transcriptomics work sheds light on stem cell features in the other major human neurogenic zone, the subventricular zone (SVZ).^98^ This study found several different stem and progenitor populations including two types of radial glia-like cells.^98^

We believe that the field will greatly benefit from transcriptomic studies in SAMP8 mice. Notably, there are currently no known scRNAseq or snRNAseq datasets of SAMP8 mice. Increasingly, microglia and astrocytes are implicated in AD disease progression.^94,99^ Identifying if the SAMP8s have similar diseased microglial profiles as other AD models may provide another marker to measure the effectiveness of interventions. In addition, such studies may identify how subpopulations of the neurogenic lineage are affected by disease progression (eg, changes in the proportion of Ki67^+^ cells in GFAP/SOX2/MASH1/DCX fraction). This will be complementary to BrdU labeling, which may vary across studies depending on the regimens employed.

### The effects of nutraceuticals on SAMP8 mice

We have used SAMP8 mice to assess the effects of nutraceuticals on the AD-like symptoms of amyloidogenesis, inflammation, memory, and adult neurogenesis.^5,77,100^ It has long been shown that nutraceuticals and their derived components, such as polyphenols and flavonoids, can reduce aspects of AD-like pathology in SAMP8 mice. For instance, long-term administration of green tea-derived catechins prevents spatial learning and memory deficits in SAMP8 mice by reducing Aβ_1-42_ oligomers and upregulating synaptic plasticity-related proteins such as BDNF and CaMKII in the hippocampus.^101^ In addition, flavonoid fisetin treatment in SAMP8 mice reduced cognitive dysfunction by increasing proteins related to stress (heat shock proteins) and inflammation (JNK and GFAP).^102^ Administration of nobiletin, a polymethoxylated flavone from citrus peels, increases glutathione peroxidase and manganese superoxide dismutase activity involved in oxidative stress in SAMP8 mice. It also decreases Tau phosphorylation in the hippocampus, thereby rescuing memory and context-dependent fear memory deficits.^103^ TCQA administration to SAMP8 mice ameliorated oxidative stress.^12^ Additionally, silibinin, a chemical found in milk thistle reduced IL-4 and IL-6 pro-inflammatory cytokines in SAMP8 mice.^104^

Our previous studies have reported that administration of nutraceuticals not only alleviates AD-like pathologies in SAMP8 mice but also promotes neurogenesis. For example, we have reported that TCQA treatment prevents and ameliorates learning and memory deficits via promoting neurogenesis in the hippocampal dentate gyrus,^5^ and that grape peel extract treatment reduces Aβ_42_ and inflammatory cytokines (TNF-α and IL-6) in the mouse brain.^6^ Furthermore, treatment of the extract of aurantiochytrium, which is a microalgae, increases the number of BrdU + GFAP + stem cells and BrdU + NeuN + mature neurons in the hippocampal dentate gyrus.^21^ Notably, SAMP8 mice given these extracts or compounds significantly improved in behavioral and cognitive tests.^5,12,13,104^

## Conclusion

Here, we highlight the SAMP8 sporadic AD model as one that recapitulates AD histopathology, including amyloid deposition, hyperphosphorylated Tau, neurogenic defects, glial cell activation, and increased ROS production. Currently, most studies using the SAMP8 model have focused on behavioral and neurogenic changes. As the importance of neuroinflammation in AD is increasingly realized, we believe that future work would benefit from including measures of glial cell activation and/or gene expression. Additionally, in-depth characterization of the nature of amyloid and tau pathology is currently lacking. We believe that the transcriptomics of the SAMP8 model is currently understudied, and future studies will bring a clearer understanding of AD and delineate the cellular mechanisms that are targeted by therapeutic interventions. While the SAMP8 model does not benefit from the straightforward genetics of familial AD models, this could further the current understanding of AD, potentially generating new genetic targets of AD. Comparison of SAMP8 studies with patient studies will determine which of the identified genes and pathways are pertinent to human AD.

## Data Availability

No new data were generated or analyzed in support of this research.
